# Usability and Quality of UCISAB, a Clinical Decision Support System for Optimization of Analgesia and Sedation Infusions in the ICU: A Multicenter, Cross-Sectional, Mixed-Methods Usability Evaluation in Colombia

**DOI:** 10.3390/jcm15187191

**Published:** 2026-09-16

**Authors:** German David Castillo-Suárez, Manuel Mena, Nadia-Juliana Proaños, Peter Vergara, Cinthya Galindo, Martha Ximena León, Fernando Ríos Barbosa, Rosa-Helena Bustos

**Affiliations:** 1 PGY-3 at the Clinical Pharmacology Department, Faculty of Medicine, Universidad de La Sabana, Campus del Puente del Común, Km 7 Autopista Norte de Bogotá, Chía 250001, Cundinamarca, Colombia; germancasu@unisabana.edu.co; 2 Evidence-Based Therapeutics Group, Department of Clinical Pharmacology, Faculty of Medicine, Universidad de La Sabana and Clinica Universidad de La Sabana, Campus del Puente del Común, Km 7 Autopista Norte de Bogotá, Chía 250001, Cundinamarca, Colombia; manuelmega@unisabana.edu.co (M.M.); pvergar6@hotmail.com (P.V.); cinthyakg@gmail.com (C.G.); 3 Doctoral Program in Biosciences, Faculty of Medicine and Faculty of Engineering, Universidad de La Sabana, Campus del Puente del Común, Km 7 Autopista Norte de Bogotá, Chía 250001, Cundinamarca, Colombia; 4 Department of Epidemiology, Faculty of Medicine, Universidad de La Sabana, Campus del Puente del Común, Km 7 Autopista Norte de Bogotá, Chía 250001, Cundinamarca, Colombia; nadia.proanos1@unisabana.edu.co; 5 Anesthesia, Pain, and Palliative Care, Faculty of Medicine, Universidad de La Sabana, Campus del Puente del Común, Km 7 Autopista Norte de Bogotá, Chía 250001, Cundinamarca, Colombia; martha.leon@unisabana.edu.co (M.X.L.); fernando.rios@unisabana.edu.co (F.R.B.)

**Keywords:** decision support systems, clinical, intensive care units, analgesia, deep sedation, task performance and analysis, user-centered design, usability testing

## Abstract

**Background:** The management of sedative and analgesic infusions in intensive care units (ICUs) represents a global clinical challenge. Clinical decision support systems (CDSSs) have emerged as an approach to facilitate protocol standardization and mitigate adverse effects associated with prolonged sedation and analgesia. **Objective:** To evaluate the usability and technical quality of UCISAB, a CDSS designed to optimize sedoanalgesia in the ICU. **Methods:** A multicenter, cross-sectional, mixed-methods usability evaluation was conducted. A prototype was evaluated using the System Usability Scale (SUS) and the User Mobile Application Rating Scale (uMARS), complemented by a qualitative analysis of open-ended comments. **Results:** A total of 31 physicians participated (attending physicians and residents). The mean SUS usability score was 66.13 (SD ±21.26), categorized as “Marginal High.” On the uMARS, the median scores were 4.28 for objective quality (good), 3.75 for subjective quality (acceptable), and 4.33 for impact (good). The stated willingness to apply the tool’s recommendations in clinical practice reached 93.5%, with a median intention-to-use score (uMARS) of 4.0 (good). Exploratory analysis revealed no significant difference in SUS scores between residents and attending physicians (*p* = 0.072). In these exploratory comparisons, statistically significant differences by professional role were found only in the uMARS dimensions: objective quality (*p* < 0.001), subjective quality (*p* = 0.023), and impact (*p* = 0.036). Qualitative feedback suggested that the tab-based architecture may increase cognitive load among expert users, highlighting a need for interface simplification. **Conclusions:** UCISAB showed favorable perceived quality and a high stated willingness to use, while usability remained marginal; further interface and pharmacological refinements, together with prospective clinical validation in real-world settings, are required before routine implementation. Professional role may influence perceived quality, suggesting the potential value of adaptive interface designs; however, this association requires confirmation in future studies. Developing systems that optimize sedation and analgesia in critical care remains an unmet need in ICUs.

## 1. Introduction

Sedative and analgesic infusions are common in intensive care units (ICUs) for patients requiring invasive mechanical ventilation. Frequently administered agents include opioids (e.g., fentanyl), benzodiazepines (e.g., midazolam), and general anesthetics (e.g., propofol) [1,2].

These agents, particularly fentanyl and other opioids, exhibit complex pharmacokinetic and pharmacodynamic profiles, characterized by a context-sensitive half-life, multicompartmental redistribution, and the risk of short-term adverse effects. A critical concern is acute tolerance, necessitating dose escalation to maintain sedation and analgesia targets. This process may precipitate iatrogenic opioid withdrawal syndrome (IOWS), with a reported incidence of 32–44% during opioid tapering in the critically ill. Despite diagnostic challenges in adults due to the lack of validated instruments, protocolized tapering of opioid infusions remains imperative in clinical practice [1,2,3,4].

An international multicenter study evaluated the availability and implementation of opioid weaning protocols in critical care. The results revealed that only 39% of ICUs utilized weaning protocols, which were implemented in only 12% of patients. Furthermore, only 10% of ICUs possessed specific IOWS protocols, applied to just 0.6% of affected patients [1].

Prolonged opioid weaning (>72 h) is associated with an increased ICU length of stay, delayed extubation, a higher risk of healthcare-associated infections, and increased mortality [4]. Consequently, protocols incorporating strategies such as methadone rotation have been proposed to mitigate and treat IOWS, demonstrating reduced time to extubation [5,6]. Systematic standardization of these protocols has significantly decreased IOWS incidence in pediatric populations [7].

This gap between evidence and practice underscores the need for a clinical decision support system (CDSS). These are information technologies that provide patient-specific processed data to assist healthcare professionals in clinical care. Rather than replacing clinical judgment, CDSS provides insights and analytics for more informed decision-making. These systems typically utilize logic-based rules derived from scientific literature, which are triggered by patient data to generate personalized recommendations [8].

In critical care, the effectiveness of CDSS has been documented mainly for ventilator weaning, where these systems have shortened the time to extubation [9] and reduced weaning duration and ICU length of stay [10]. Sedoanalgesia tapering is an equally critical process that, unlike ventilatory weaning, requires the integration of several sources of information—sedation and pain targets, the pharmacokinetic behavior of the infused agents, and the risk of withdrawal—within a single decision [11].

Evidence on medication support for sedation and analgesia points in the same direction. Structured, algorithm-based management of analgesic and sedative infusions has been shown to modify clinically relevant outcomes: the replacement of fentanyl infusions by enteral methadone reduced the weaning time from mechanical ventilation in a randomized controlled trial [6], and the implementation of an algorithm for tapering analgosedation reduced iatrogenic withdrawal syndrome in pediatric intensive care [7]. Evidence-based recommendations for the management of sedoanalgesia in critically ill adults are also available [5]. The limitation is therefore not the absence of evidence but rather its delivery at the bedside, where the availability and application of such protocols remain low [1].

However, the evidence base for CDSS in critical care has been built around clinical processes other than sedation and analgesia. Published systems and usability evaluations have targeted ventilator weaning [9,10], continuous kidney replacement therapy [12], early recovery after traumatic brain injury [13], and mortality or severity score calculation in smartphone applications [14]. For sedoanalgesia, the resources available at the bedside are largely general-purpose medical calculators that resolve isolated variables and do not integrate, within a single workflow, the target RASS and CPOT scores, the drug concentrations and mixtures locally available, the duration and rate of the ongoing infusion, and the stratification of IOWS risk required for a protocolized taper. To the best of our knowledge, no CDSS specifically devoted to the initiation, titration, and weaning of analgesic and sedative infusions in the adult ICU has been formally evaluated for usability and technical quality.

This context highlights a significant research gap previously explored by the Therapeutic Evidence research group at Universidad de La Sabana. Their study identified clinicians’ pharmacological and pharmaceutical requirements for ICU sedoanalgesia, revealing that 95% of intensivists consider technological tools necessary to optimize the adjustment, titration, and dose calculation of opioid infusions [15]. UCISAB was developed to address this specific unmet need, bringing together in a single patient-specific workflow the initiation and titration of infusions, protocolized tapering, and IOWS risk stratification, based on the sedation and analgesia recommendations of evidence-based guidelines [5] and on the drug presentations available in Colombian ICUs.

Healthcare professional acceptance is a critical success factor for CDSS. Research indicates that adoption depends on strictly aligning evidence-based recommendations, alerts, and diagnostic suggestions with user requirements [16]. Moreover, the information management cycle (input, processing, and output) must integrate seamlessly into the clinical workflow to prevent added cognitive load or alert fatigue [17].

Consequently, before clinical implementation, health technologies must be aligned with user needs through robust evaluation methods to ensure optimal usability and quality standards [18]. Therefore, the present study aims to evaluate the quality and usability of the initial version of a CDSS focused on optimizing sedation and analgesia infusions in the intensive care unit. As a secondary, exploratory objective, differences in perceived usability and quality according to professional role (residents versus attending physicians) were examined. Given the available sample size, this comparison was planned as exploratory and hypothesis-generating rather than confirmatory.

## 2. Materials and Methods

### 2.1. Study Design

A multicenter, cross-sectional, mixed-methods usability evaluation was conducted at two hospital centers in Colombia: Clínica Nueva (Bogotá) and Clínica Universidad de La Sabana (Chía, Cundinamarca). Data collection took place between February and April 2026.

### 2.2. UCISAB CDSS Description

UCISAB is a CDSS developed by the Therapeutic Evidence research group at Universidad de La Sabana. The development process followed a design thinking framework, a human-centered methodology that prioritizes problem-solving based on end-user needs and preferences [19]. The CDSS architecture was implemented using Zoho Creator^®^ 1.0, a low-code development platform that enables the creation of custom desktop and mobile applications without requiring advanced programming or coding expertise. Consequently, healthcare professionals with minimal technical knowledge can design and implement functional mobile applications tailored to their specific requirements. This approach promotes technological democratization within the healthcare sector, allowing innovation to emerge directly from clinical staff needs [20].

The clinical algorithms underlying the CDSS (including dosage recommendations for initiation, progressive weaning protocols, and diagnostic criteria for IOWS) were synthesized from evidence-based guidelines and key clinical studies. Specifically, these parameters integrate the sedation and analgesia frameworks established by Celis-Rodríguez et al. [5] and the pharmacological evidence validated in recent systematic reviews [21,22,23] (Supplementary Material Section S1).

**Figure 1:**
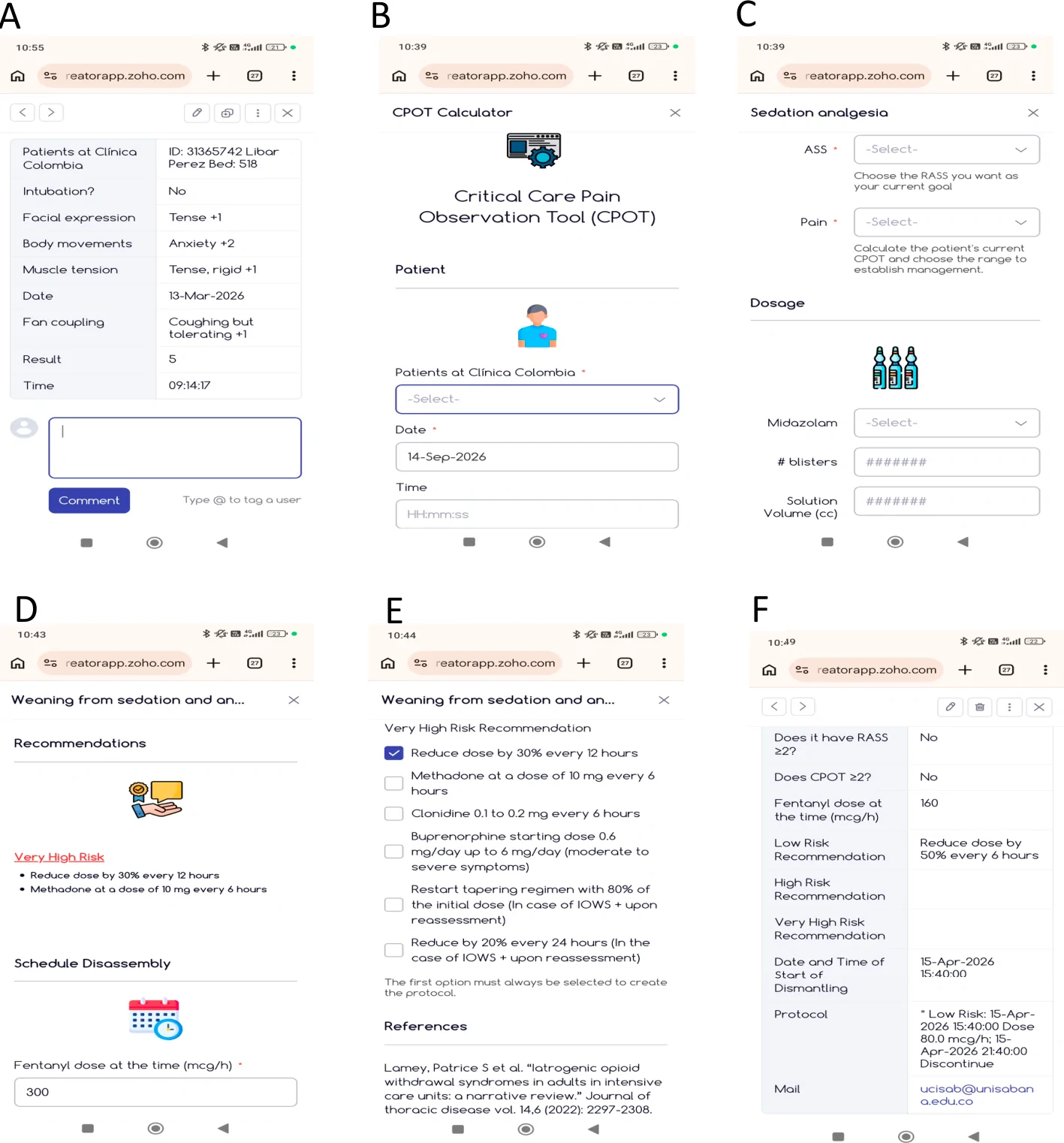
Visual layout and key functional modules of the UCISAB mobile application. (**A**,**B**) Patient profile management and local record generation. (**C**) Sedation and analgesia initiation module, including RASS/CPOT input fields and initial dosage calculator. (**D**–**F**) Progressive tapering and IOWS risk stratification interface, providing protocolized weaning recommendations. CPOT: Critical Care Pain Observation Tool; RASS: Richmond Agitation–Sedation Scale; IOWS: iatrogenic opioid withdrawal syndrome.

The tool is available in both mobile and desktop versions. The system workflow is divided into three main phases:

1. Profile Management: Upon initiation, the application prompts the creation of a patient profile, generating a local record that allows for personalized data consultation, modification, or deletion (Figure 1A,B).

2. Sedation and Analgesia Initiation Module: After selecting this option, the interface requires the input of objective clinical variables, including the target Richmond Agitation–Sedation Scale (RASS) score and the current Critical Care Pain Observation Tool (CPOT) score, as well as available drug concentrations and mixtures. Based on these data, the system calculates and recommends initial infusion dosages (Figure 1C).

The clinical rules underlying these modules were operationalized as follows. Since the version evaluated corresponds to a minimum viable product, algorithms were implemented for the three sedatives most frequently used in our clinical context—midazolam, propofol, and ketamine—and for fentanyl, the opioid most frequently used in this setting. Sedative infusion rates are titrated to the target RASS score entered by the user, within the dosing ranges reported by Devlin et al. [24]: a target RASS of −1 to −5, midazolam ranges from 0.04 to 0.12 mg/kg/h, propofol from 0.3 to 4 mg/kg/h, and ketamine from 1 to 2 mg/kg/h, with no infusion recommended for a target RASS of 0. Analgesia is titrated to the CPOT score using the ranges reported by the same source: 1 µg/kg/h of fentanyl for mild pain (CPOT below 2), 3 µg/kg/h for moderate pain (CPOT 2 to 4), and 5 µg/kg/h for severe pain (CPOT above 4). Within these ranges, the specific value assigned to each RASS and CPOT level was defined pragmatically by author consensus for this prototype. The module operates as a calculator intended to provide a supporting margin, and the final dose remains a decision of the treating physician. The complete dose table is provided in Table S4 (Supplementary Material Section S1).

The rules of the IOWS module were adapted from evidence syntheses and published protocols [22,23]. Because the available evidence was heterogeneous at the time of development, a rule consistent with the reported protocols was defined by author consensus. Five clinical variables are used to classify the patient into one of four risk levels: duration of opioid infusion (less than 7 days or 7 days or more), presence of risk factors for iatrogenic withdrawal, presence of two or more withdrawal signs, a RASS score of 2 or higher, and a CPOT score of 2 or higher. The 7-day threshold for opioid exposure was also established by consensus. Withdrawal signs were assessed using the DSM-5 criteria adapted to the local context, which have been reported as potentially useful for the diagnosis of IOWS in adult patients [22,23]. The combinations defining each risk level, together with the corresponding action taken by the system, are presented in Table S5 (Supplementary Material Section S1).

Tapering recommendations were derived from the same sources and defined by author consensus for each risk level. When no risk is identified, direct discontinuation of the infusion is proposed. At low risk, a 50% dose reduction every 6 h is recommended, with fentanyl rescue doses of 1–2 µg/kg. At high risk, a 30% reduction every 6 h is recommended, together with fentanyl rescue doses and the option of methadone (10 mg every 6 h) or clonidine, or resumption at 80% of the previous rate if symptoms reappear at reassessment. At very high risk, a 30% reduction every 12 h is recommended, with methadone (10 mg every 6 h), clonidine, or buprenorphine at an initial dose of 0.6 mg/day; a 20% reduction every 24 h or a return to 80% of the previous dose may also be considered if IOWS is confirmed. No formal external validation of these rules against a reference standard was carried out prior to the usability evaluation.

3. Progressive Tapering and Risk Stratification Module: For the weaning process, the system prompts for data such as previous fentanyl infusion duration, current infusion rate, prevailing RASS score, and IOWS risk factors. Subsequently, the application performs a withdrawal risk stratification and provides specific recommendations for a protocolized reduction (Figure 1D–F).

**Figure 2:**
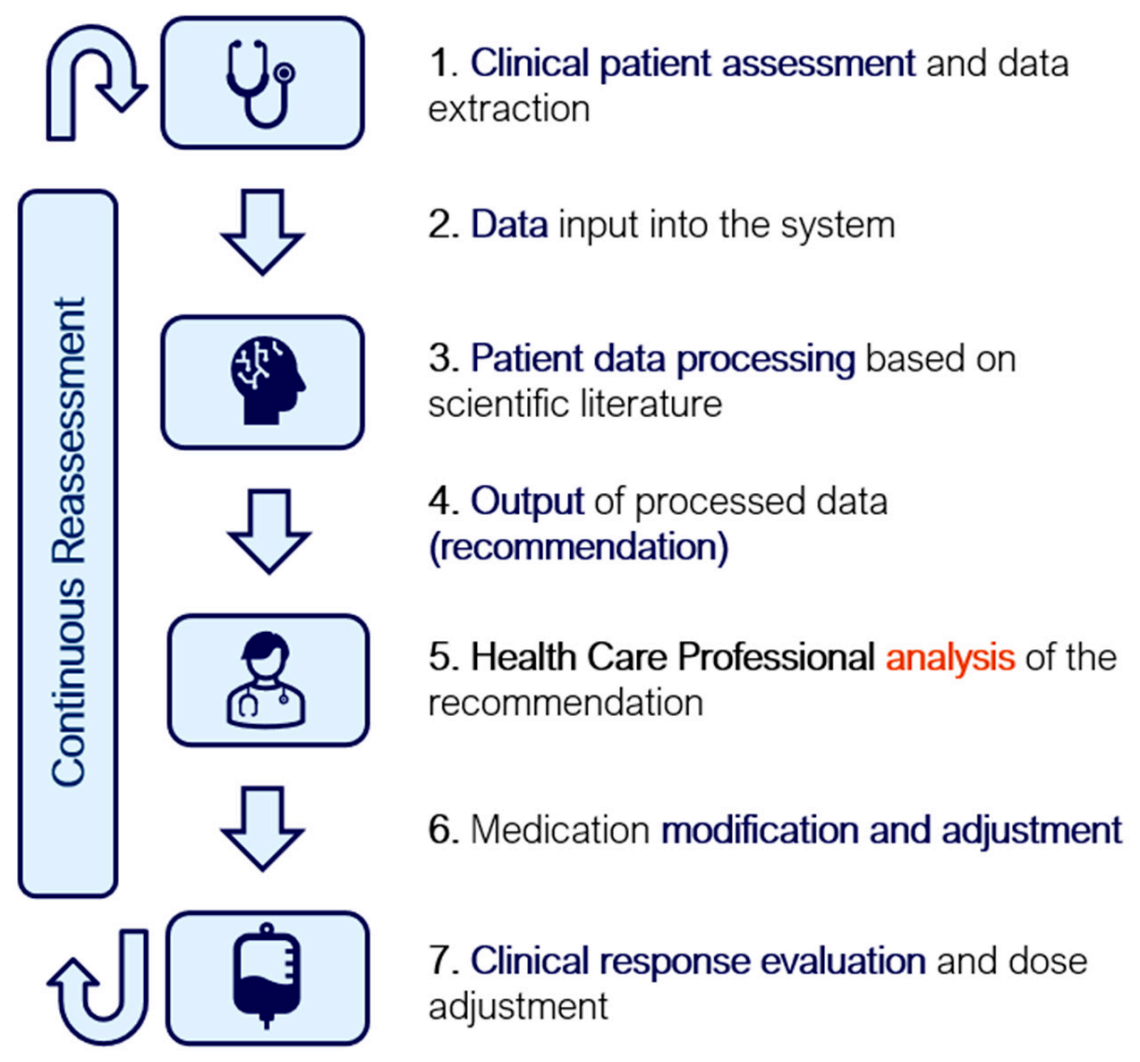
UCISAB clinical workflow and decision-making process. The diagram illustrates the dynamic integration of the system into the ICU routine, emphasizing the cycle of data processing and continuous patient reassessment.

UCISAB was designed to integrate dynamically into the ICU workflow, promoting continuous patient reassessment following each intervention to monitor the clinical effect of the suggested recommendations (Figure 2).

### 2.3. Usability and Quality Assessment Tools

The System Usability Scale (SUS) was developed by John Brooke in 1986 for electronic system evaluation. It is currently one of the most robust and widely used instruments for measuring the usability of digital health applications [25], reporting high internal consistency, with Cronbach’s alpha coefficients ranging from 0.85 to 0.91. The present study utilized the Spanish-validated version by Del Rocío-Sevilla et al. [26].

The SUS consists of a 10-item five-point Likert scale (ranging from “strongly disagree” to “strongly agree”), featuring statements with alternating positive and negative valences to minimize acquiescence bias. The global score is calculated using the following algorithm: for odd-numbered items (positive statements), 1 is subtracted from the selected value; for even-numbered items (negative statements), the selected value is subtracted from 5. The sum of these scores is multiplied by 2.5 to achieve a final range of 0–100. According to established benchmarking criteria, a score ≥ 85 reflects excellent usability, while scores between 68 and 84 indicate good or acceptable usability [26,27]. Despite the development of the Healthcare Systems Usability Scale (HSUS) to address specific dimensions such as patient safety, the standard SUS was selected due to the absence of a validated Spanish version at the time of this study [28].

The user version of the Mobile Application Rating Scale (uMARS) is a derivative of the original Mobile Application Rating Scale (MARS) developed and validated by Stoyanov et al. [29,30]. Currently, this instrument is established as the second most frequently utilized scale in medical literature for evaluating health applications, preceded only by the SUS [25]. The present study employed the Spanish-validated version, which demonstrates robust internal consistency with Cronbach’s alpha coefficients of 0.89 for objective quality, 0.67 for subjective quality, and a global value of 0.90 [31].

The uMARS is a 26-item questionnaire, with all items rated on a 5-point Likert scale ranging from 1 (inadequate) to 5 (excellent), organized into three components: (a) objective quality (16 items), comprising four subscales—Section A: engagement (items 1–5), Section B: functionality (items 6–9), Section C: aesthetics (items 10–12), and Section D: information (items 13–16)—each scored as the mean of its items, with the overall objective quality score calculated as the mean of the four subscale scores; (b) subjective quality (Section E, 4 items, 17–20), calculated as the mean of four items assessing the likelihood of recommendation, anticipated future use, willingness to pay, and overall star rating; the recommendation and anticipated future use items were additionally examined individually and labeled as “Acceptability: Recommendation” and “Acceptability: Adoption,” respectively, following the implementation outcomes terminology applied by Rodríguez et al. [32]; and (c) perceived impact (Section F, 6 items, 21–26), calculated as the mean of its six items, assessing the tool’s capacity to influence the clinician’s awareness, knowledge, attitudes, and behavior change intention regarding the targeted health objective. All domain scores are interpreted on the same 1–5 scale (1 = inadequate, 5 = excellent), and individual items may additionally be examined separately to identify specific strengths or weaknesses of the tool [30,31].

### 2.4. Participants and Eligibility

Healthcare professionals (attending physicians and residents) affiliated with intensive care units, with at least one year of experience in the management of sedative and analgesic infusions in critical care settings, were included. Exclusion criteria included prior participation in any phase of the CDSS design or development, as well as being a general practitioner or a professional not currently enrolled in a medical–surgical residency program. Participants were recruited using a non-probabilistic convenience sampling method, complemented by peer referral. Attending physicians and residents present in the intensive care units of each participating center during the study period were invited to participate through direct electronic invitations (email and instant messaging) and, in some cases, through referral by collaborating specialists at each site. A total of 42 physicians were invited to participate; of these, 37 initiated and returned an evaluation, corresponding to a response rate of 88.1% (37/42), defined as the proportion of invited physicians who returned the data collection instrument, whether complete or incomplete. Of these 37 records, two were incomplete, and four had been completed by general practitioners, who did not meet the eligibility criteria; the remaining 31 records met all inclusion criteria and were included in the final analysis. Regarding distribution by center, 22 participants were recruited from Clínica Universidad de La Sabana (Chía) and 9 from Clínica Nueva (Bogotá). Participation was strictly voluntary, and each professional provided written informed consent prior to data collection.

The sample size was calculated to estimate the mean SUS and uMARS scores with a 95% confidence level (Z = 1.96) and a precision margin (E) of 5 points for the SUS and 0.25 for the uMARS. Calculations were based on the standard normal distribution reported in the literature for both scales, assuming a standard deviation (σ) of 12.5 points for the SUS [27] and 0.48 for the uMARS [33]. Following the formula for cross-sectional studies proposed by Charan et al. [34]:

$$n=\frac{Z^{2}.\sigma^{2}}{E^{2}\;}$$

These parameters indicated a minimum requirement of 15 subjects for the uMARS and 25 for the SUS. To account for a potential 20% attrition rate, the final recruitment target was set at 30 participants, satisfying the most stringent requirement (SUS).

### 2.5. Procedures

After confirming eligibility, participants received a detailed explanation of the study and provided written informed consent. A synchronous induction session was conducted via the Microsoft Teams^®^ platform, addressing the following: (1) the architecture and functionality of the UCISAB CDSS; (2) the clinical and pharmacological evidence supporting the system; and (3) technical training on completing the SUS and uMARS. Subsequently, participants evaluated the CDSS functions through two simulated clinical cases in a controlled environment. This stage allowed for unrestricted interaction to ensure familiarity with the tool’s interface before formal assessment. Finally, participants completed the data collection instrument hosted on the REDCap^®^ 13.1.4-© 2026, Vanderbilt University (Research Electronic Data Capture) platform.

Both simulated clinical cases were identical for all participants, ensuring standardized exposure across the sample. The cases were presented in a fixed order and represented two prototypical ICU sedoanalgesia scenarios: Case 1 required the use of the sedoanalgesia calculator module for a mechanically ventilated patient requiring deep sedation, followed by the tapering module for the same patient after prolonged fentanyl infusion with autonomic withdrawal features; Case 2 required the tapering module for a patient with chronic opioid use and risk factors for iatrogenic opioid withdrawal syndrome. For each case, participants entered the clinical data into UCISAB, reviewed the dose recommendations generated by the system, customized drug presentations and dilutions according to their institutional practice, and selected the options they would apply based on their clinical judgment. All sessions were supervised in real time (in person or remotely) by a member of the research team, who clarified questions regarding the use of UCISAB and the completion of the SUS and uMARS. Interaction with the tool was unrestricted, with no fixed time limit imposed; the complete case session lasted approximately 30 min. The complete case scenarios are provided in Supplementary Material Section S2.

Finally, the mobile and desktop versions of UCISAB were available to participants during the evaluation, and each participant was free to choose the version according to their preference and available device. The specific version used by each participant was not recorded.

### 2.6. Ethical Considerations

This research adhered to the ethical principles of the Declaration of Helsinki, Good Clinical Practice (GCP) guidelines, and Colombian regulations under Resolution 8430 of 1993. The study was classified as minimal-risk research. In compliance with Statutory Law 1581 of 2012 (Habeas Data) and Regulatory Decree 1377 of 2013, personal data protection and the confidential handling of sensitive information were guaranteed. The protocol received formal approval from the Institutional Review Board (IRB)/Ethics Committee of the Clínica Universidad de La Sabana (4 November 2025).

### 2.7. Statistical Analysis

Statistical analysis and data visualization were performed using JASP^®^ (version 0.96). To ensure reproducibility and rigor, an independent verification of all calculations was conducted by a second researcher using STATA^®^ (version 17.0). Internal consistency of the SUS and uMARS was evaluated using Cronbach’s alpha (α). Data distribution was determined using the Shapiro–Wilk test. Normally distributed quantitative variables were reported as mean and standard deviation (SD), while non-normally distributed variables were expressed as median and interquartile range (IQR). Exploratory comparisons between groups were performed using independent samples *t*-tests for parametric data and the Mann–Whitney U test for non-parametric distributions. All tests were two-tailed, and statistical significance was set at *p* < 0.05. For each comparison, effect sizes were reported alongside *p*-values: Cohen’s d with 95% confidence intervals for the SUS and rank-biserial correlation with 95% confidence intervals for the uMARS dimensions, as effect sizes are less dependent on sample size and more informative than *p*-values alone in exploratory analyses with limited power.

Complementing the quantitative approach, open-ended feedback from the participants underwent a six-phase reflexive thematic analysis following the Braun and Clarke (2006) framework [35]. The analysis was conducted by a single coder (the principal investigator, a physician with training in clinical pharmacology), consistent with the reflexive approach, in which the researcher’s interpretative engagement is regarded as a resource rather than a source of bias, and inter-coder agreement is not treated as a required validity criterion. The process comprised: (1) familiarization with the data through repeated reading of all responses; (2) generation of initial codes applied systematically across the dataset; (3) collation of codes into candidate themes; (4) review of themes against the coded extracts and the full dataset; (5) definition and naming of final themes; and (6) selection of representative quotes for reporting. Non-substantive responses (e.g., single punctuation marks or generic praise without argumentative content) were excluded.

## 3. Results

Between February and April 2026, 42 physicians were invited, and 37 returned an evaluation of the UCISAB CDSS. Six of these records were excluded: two were incomplete, and four had been completed by general practitioners, who were not eligible. The final analytical sample therefore consisted of 31 evaluations (*n* = 31). No missing data were present in the analyzed sample; all 31 participants completed all items of both the SUS and uMARS instruments, so no imputation or missing-data handling procedures were required.

### 3.1. Participant Characteristics

**Figure 3:**
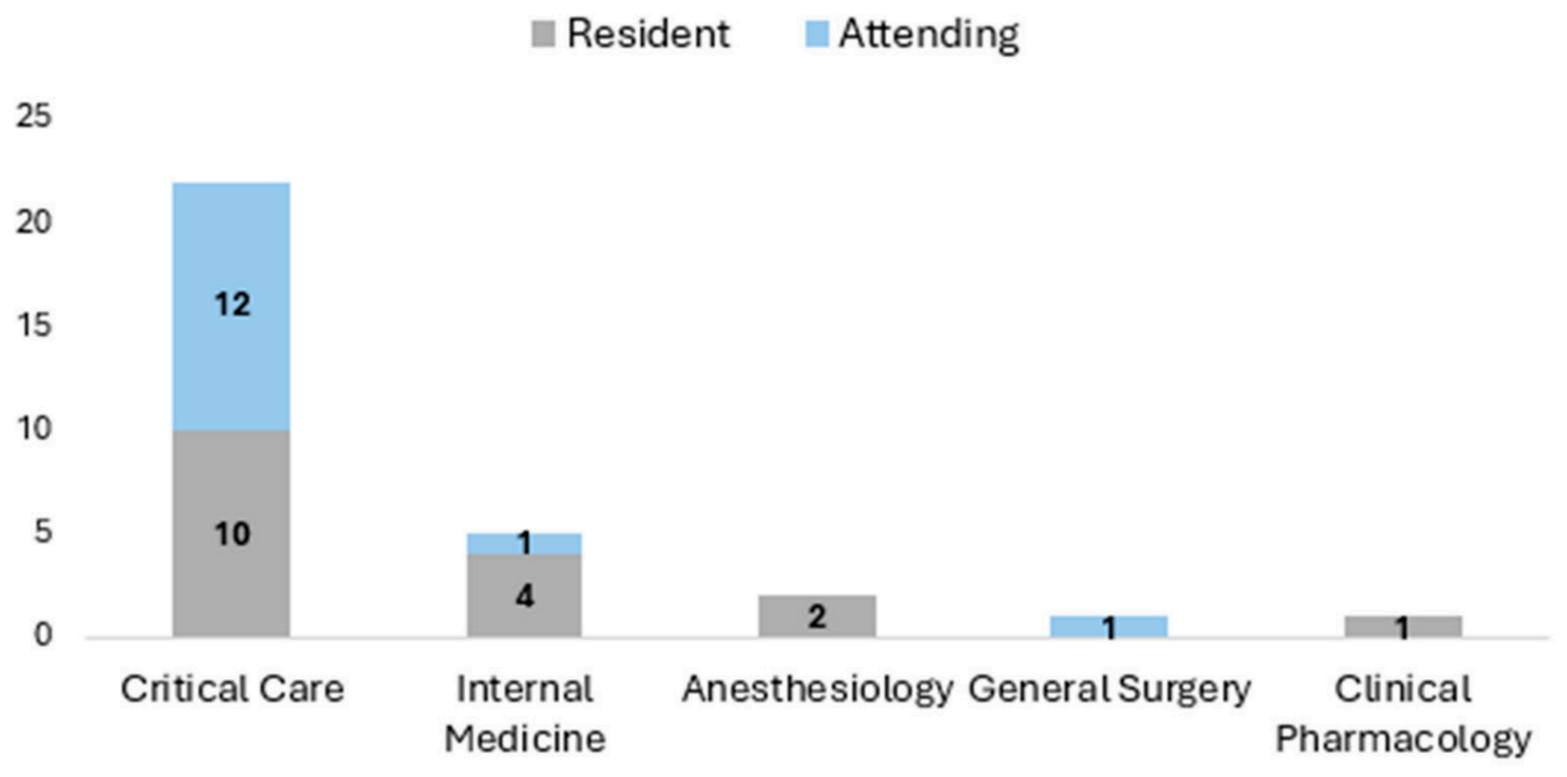
Distribution of participants by medical specialty and professional role.

As detailed in Table 1, 48.4% of participants were male, and 45.2% were attending physicians. Notably, a significant majority (71.0%) belonged to the Critical Care specialty. Regarding clinical experience, 19.4% had more than 10 years of practice, while 22.6% had between 6 and 10 years. The mean age was 34.7 ± 7.3 years. Additionally, 35.5% reported prior experience with medical applications. Within the Critical Care subgroup, 45.0% were residents and 55.0% were attending physicians; among the latter, 86.0% were critical care specialists and 14.0% belonged to other specialties, such as General Surgery and Internal Medicine (Figure 3).

### 3.2. Instrument Reliability and Data Distribution

Table 2 presents the internal consistency, yielding a Cronbach’s alpha of α = 0.883 for the SUS and an α = 0.943 for the objective quality subscale of the uMARS. In the dimensional analysis of the uMARS, all subscales achieved α values greater than 0.84. The Shapiro–Wilk test and kernel density analysis (Supplementary Material, Figure S1) for the overall usability score (SUS) revealed a normal distribution (*p* = 0.451), whereas the uMARS objective quality exhibited a non-normal distribution (*p* = 0.019). This finding justified the use of non-parametric statistics for correlation analysis.

### 3.3. Quality and Usability Assessment

Table 3 and Table 4 summarize the results for quality and usability. The uMARS evaluation yielded good perceived objective quality with a median of 4.28 (IQR: 3.57–4.63), acceptable subjective quality of 3.75 (IQR: 3.12–4.12), and a high perceived impact of 4.33 (IQR: 4.00–5.00). All objective subscales remained within the “good quality” range: engagement (4.20; IQR: 3.30–4.60), functionality (4.25; IQR: 3.75–4.75), aesthetics (4.33; IQR: 3.33–4.67), and information (4.50; IQR: 4.00–5.00). Regarding usability, the mean SUS score was 66.13 (SD = 21.26). This value places the UCISAB CDSS slightly below the traditional threshold for adequate usability (68.0) established in the literature.

### 3.4. Stated Willingness to Use and Qualitative Feedback

**Figure 4:**
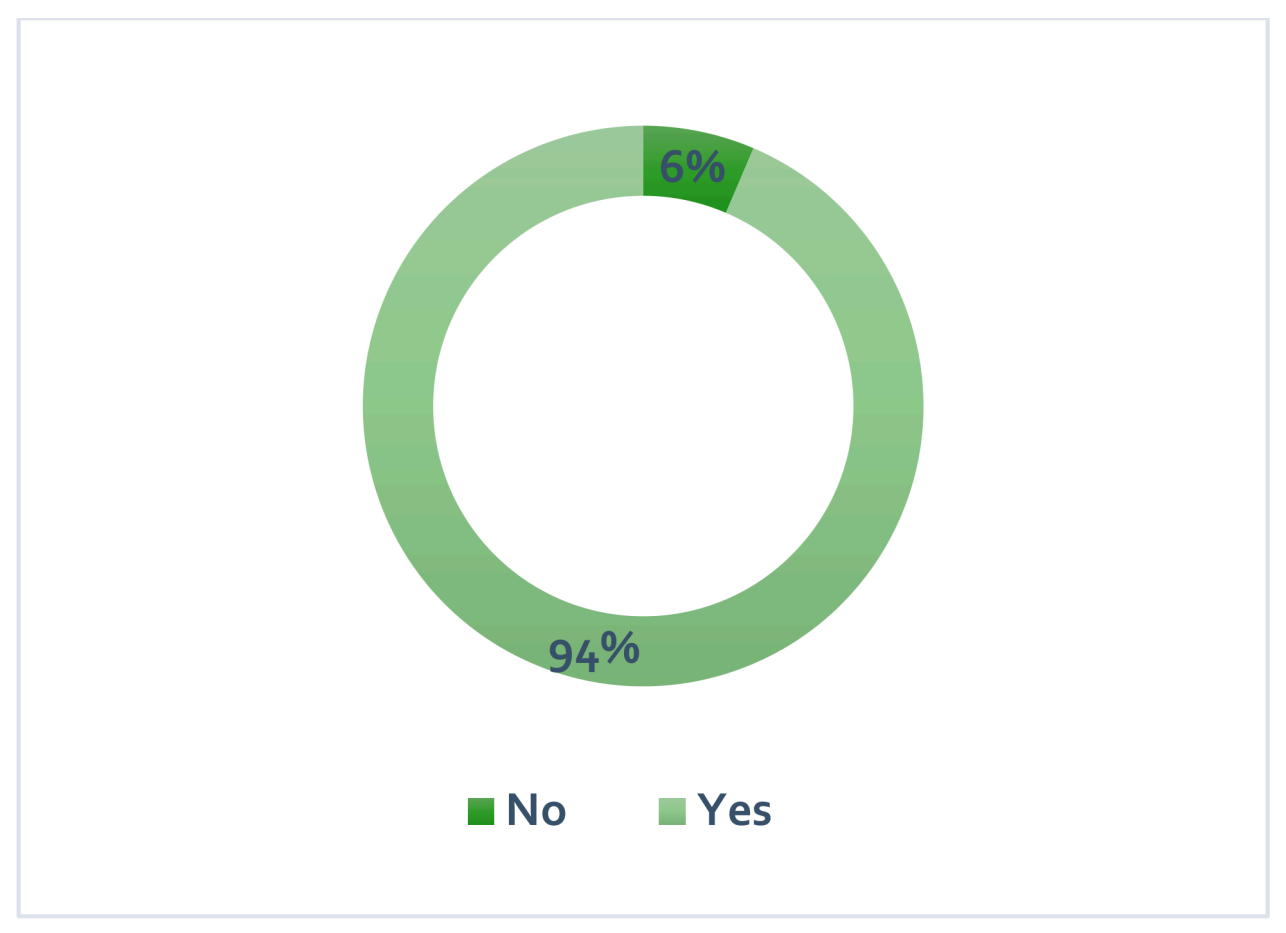
Acceptance frequency of UCISAB results for real clinical practice among medical personnel (*n* = 31). A total of 93.5% of participants (*n* = 29) reported that they would apply the tool’s results in daily clinical practice. This item reflects stated willingness to use only; the safety of the recommendations generated by UCISAB was not assessed in this study.

Despite the usability score, participants’ stated willingness to apply the tool’s recommendations in a real clinical scenario was remarkably high: 93.5% (*n* = 29) answered affirmatively to the question, “*If the simulated patients were real, would you apply the results obtained in clinical practice?*” (Figure 4). For the open-ended feedback, non-substantive responses were excluded (e.g., single punctuation marks or generic praise without argumentative support), resulting in 16 substantive units of analysis (62.5% specialists, *n* = 10; 37.5% residents, *n* = 6). Thematic analysis identified three core themes: (1) clinical utility: focus on care impact and evidence-based support; (2) interface and user experience (UI-UX) challenges: navigation and interface optimization opportunities; and (3) algorithmic refinement and pharmacological depth: where adjustments in dosing and expansion of the drug formulary were suggested (Table 5).

## 4. Discussion

This study represents the first formal evaluation of the usability and technical quality of a CDSS designed to optimize sedation and analgesia in the ICU (UCISAB) in Colombia. This multicenter, cross-sectional, mixed-methods usability evaluation integrated the uMARS and SUS with qualitative analysis and assessed stated willingness to apply the tool’s recommendations using simulated clinical cases. The evaluation was carried out remotely, in a controlled and simulated environment, and not during the care of real patients; the findings therefore describe how the system was perceived by clinicians under simulation and not its performance in routine intensive care practice.

Findings revealed a mean SUS score of 66.13, classified as “Marginal High” according to the adjective rating scale by Bangor et al. Although this score is slightly (1.87 points) below the literature-recommended threshold of 68 for “good” usability [26,36]. These results must be interpreted within the context of the high intrinsic complexity of CDSS in critical care environments, where the information load is substantially higher than in general-purpose applications.

When contrasting these results with those of other ICU-based CDSS, higher scores are observed in specific prototypes. For example, the evaluation of a system for renal replacement therapy reported a mean of 87.5 (excellent) [12]; however, that study utilized a small sample (*n* = 12) of nursing and critical care professionals with a mean expertise exceeding 14 years, which may have facilitated technical interaction. Similarly, a neurocritical rehabilitation CDSS obtained a SUS score of 83.6, using a sample of intensive care, nursing, and respiratory therapy professionals (*n* = 18), highlighting that a modular and structured interface is key to reducing cognitive load [13]. In contrast, the UCISAB evaluation involved a larger and more heterogeneous sample (*n* = 31) that included medical residents, providing a more realistic perspective of usability challenges in a diverse clinical setting.

Regarding technical quality indicators, UCISAB ranked within the “good quality” range for the objective quality and impact dimensions, while the subjective quality dimension was classified as “acceptable.” In contrast with the literature on digital tools in critical care settings, Fijačko et al. (2021) evaluated 21 applications designed for SOFA and APACHE II score calculations. In their research, conducted by nursing staff with over five years of ICU expertise, the global mean score was 3.66 (SD = 0.65), identifying the *MDCalc Medical Calculator* as the highest-rated benchmark at 4.75 points [14].

Notably, UCISAB outperformed the average of the commercial tools analyzed by Fijačko et al. [14], despite those evaluations being performed by personnel with an expertise profile equivalent to our attendings. While *MDCalc* remains the benchmark with the highest score, the 4.50 median obtained by UCISAB in the information dimension reflects high perceived information quality, comparable to that reported for leading medical calculators in commercial app repositories. This finding should be interpreted as a perception-based comparison rather than evidence of validated algorithmic performance, given that uMARS assesses user-perceived quality rather than algorithmic accuracy.

The triangulation of qualitative feedback with SUS and uMARS scores identified three determinant axes for system optimization: (1) interface and user experience (UX); (2) algorithmic refinement and pharmacological depth; and (3) clinical utility and acceptability.

### 4.1. Interface and User Experience (UX)

Qualitative findings highlight that UCISAB’s primary challenges reside in its graphic design and aesthetics. Evaluators suggested integrating enhanced visual content to support various windows and functionalities, thereby optimizing navigation flow. Regarding information architecture, a critical recommendation emerged: transitioning from a tab-based layout to a sequential workflow. While tab-based designs allow for customization, users reported that this fragmentation creates a sense of “disconnection” within the clinical workflow and perceived it as potentially increasing cognitive load. This observation aligns with Nielsen’s usability heuristics, specifically the principle of “visibility of system status”. A fragmented interface forces clinicians to exert additional working memory effort to recall their current stage within the sedation protocol, rather than receiving continuous visual feedback to guide real-time decision-making [37].

In contrast with the uMARS results, the functionality and aesthetics dimensions achieved satisfactory scores (medians: 4.25 [IQR 3.75–4.75] and 4.33 [IQR 3.33–4.67], respectively). These ratings reflect a positive perception of the system’s functional performance and visual design, rather than a limitation in its perceived clinical utility. However, there is a clear convergence with the mean usability score (66.13), where the SUS captured the “operational friction” described by experts. This friction was attributed by participants to interface fragmentation and the absence of sequential iconography, both of which were perceived as potentially contributing to cognitive burden; cognitive load was not directly measured in this study. Collectively, these data suggest that aesthetics in a CDSS are not merely ornamental but are perceived by users as relevant to their interaction with the system; its actual impact on clinical efficiency and on patient safety was not measured in this study.

### 4.2. Algorithmic Refinement and Pharmacological Depth

A second fundamental axis for the system’s evolution is the clinical explainability of its recommendations. While “explainability” is frequently associated with artificial intelligence, in the context of a CDSS, it implies that providing accurate data is insufficient; the system must render the underlying clinical reasoning transparent. This transparency is a critical determinant of user trustworthiness in the tool [38]. Qualitative feedback from evaluators (who requested the integration of comprehensive “clinical guidance” beyond simple dosage calculation) highlights that visibility of bibliographic sources and clarity in algorithmic logic are priority areas for improvement [39]. These elements are essential to ensure the traceability of automated reasoning and to strengthen the technical foundation of recommendations in complex decision-making scenarios [40].

Furthermore, suggestions to integrate advanced functions, such as cumulative opioid dose monitoring and protocols for the differential diagnosis of opioid and benzodiazepine withdrawal syndromes, reveal critical gaps in the current intensivist workflow. Including these features would not only increase the clinical value of UCISAB but also address the need to adapt prototypes to the real-world demands of end-users, thereby reducing task fragmentation during clinical rounds [41].

Finally, adaptation to the clinical micro-context emerged as a decisive factor for usability. Observations regarding the need to synchronize the system with local propofol presentations and midazolam concentrations suggest that perceived CDSS usability depends on its alignment with local operational realities [11,42]. Any discrepancy between system units and those used in daily practice could impose an unnecessary cognitive load during the titration of critical medications, an aspect that merits attention in future error-risk assessments [37].

### 4.3. Information Quality and Adoption Facilitators

UCISAB’s primary strength lay in its information quality, achieving the study’s highest median score of 4.5 (IQR: 4.0–5.0). This reflects a superior perception of the system’s scientific validity and evidence-based foundations. In critical care, where patient safety is paramount, rigorous evidence traceability is a desirable feature of automated decision support tools intended for future clinical use.

High stated willingness to use further supports a positive user perception of the system. The 93.5% stated willingness to apply the tool’s recommendations in clinical practice suggests a favorable user perception of the tool as a clinical decision aid, independent of current interface limitations. This is further supported by favorable acceptability-related scores (medians of 4.00 for both peer recommendation and short-term future use intention, as these specific subjective quality items are part of the acceptability evaluation described by [32]) and a perceived impact score of 4.33. This aligns with Human Factors Engineering (HFE) principles: a CDSS must act as a workflow facilitator rather than a cognitive burden. Meta-analytical data show that only 34.2% (95% CI: 23.2–47.1%) of global CDSS achieve favorable acceptability, primarily due to poor usability [40]. Similarly, systematic reviews assessing the long-term evolution of CDSS (from 6 months to 5 years) confirm that ease of use and minimal time investment by clinicians are the main facilitators of success [41].

### 4.4. Exploratory Analysis

**Figure 5:**
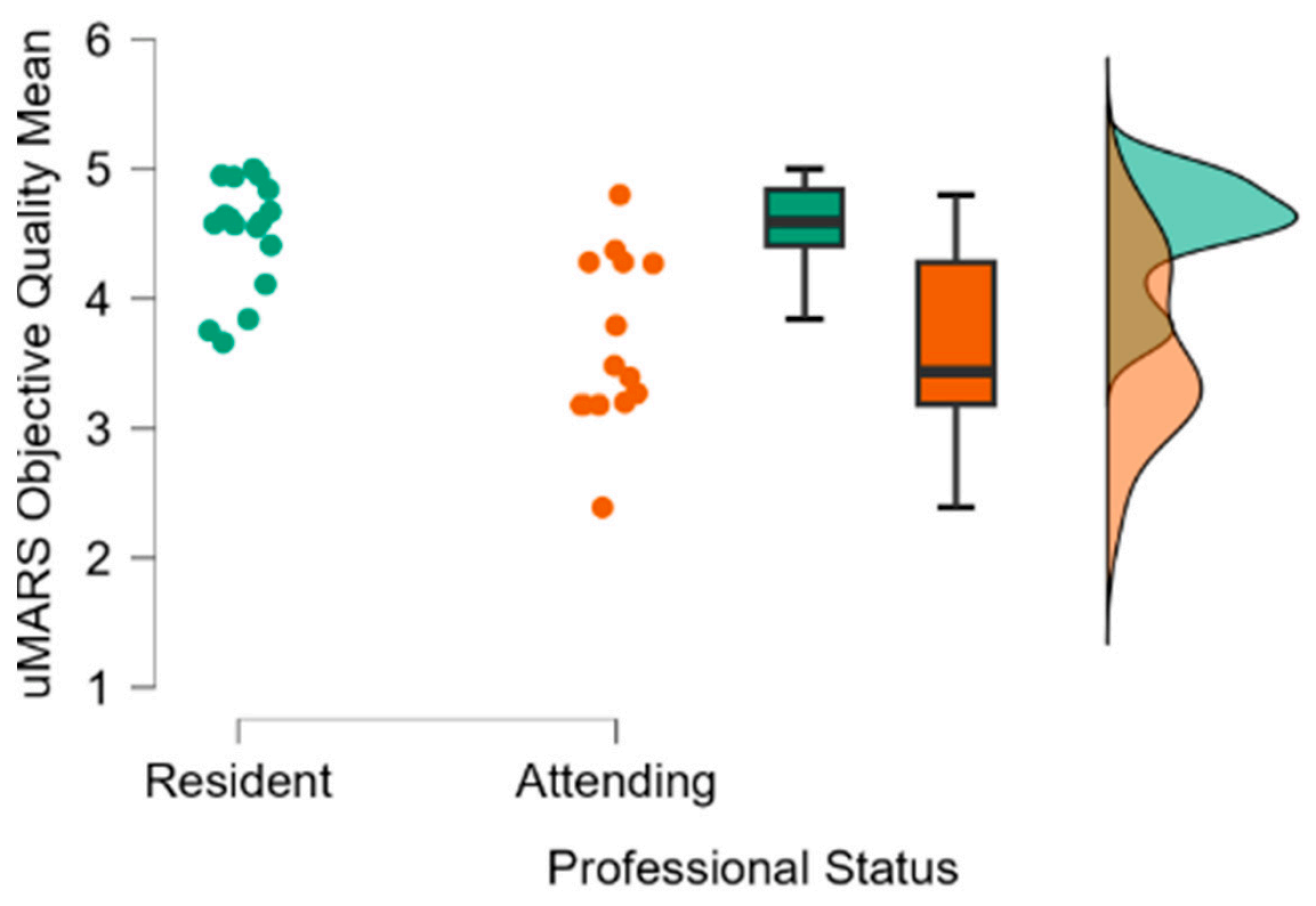
Raincloud plot visualization of the overall objective quality (uMARS) scores by professional role. Note the tighter convergence of high scores within the resident group compared to the greater dispersion observed among attendings.

**Figure 6:**
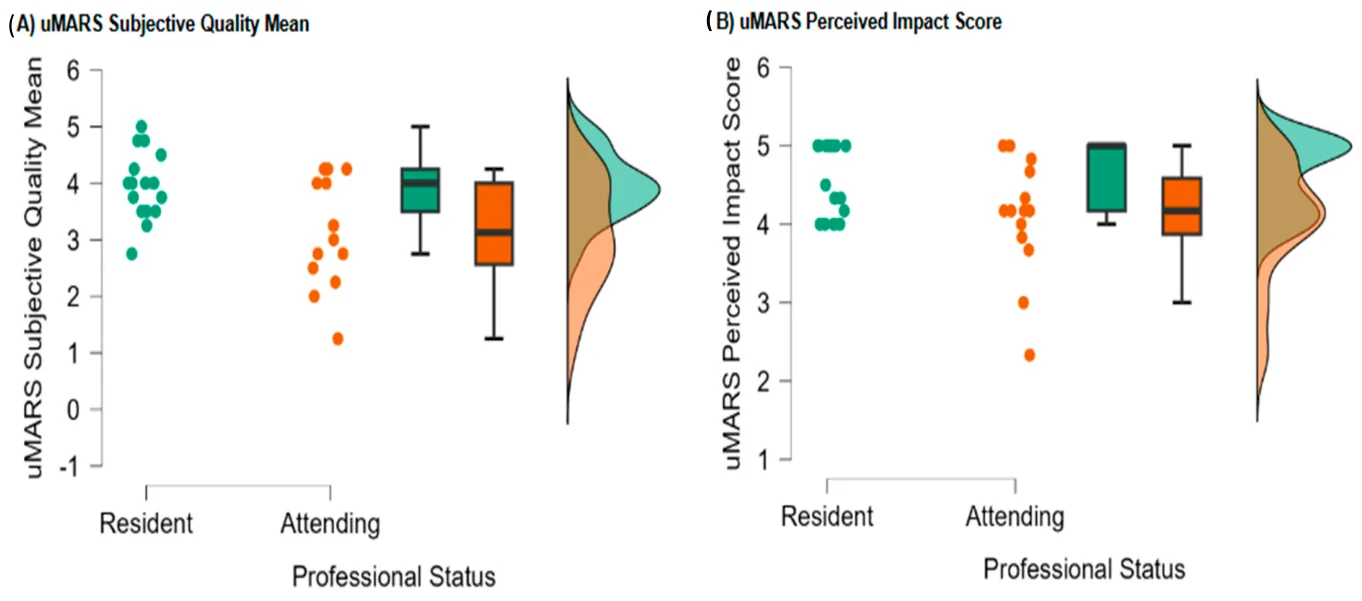
Raincloud plot visualization of (**A**) mean subjective quality and (**B**) perceived impact scores (uMARS) by the evaluators’ professional role. Note the significant difference in subjective quality (*p* = 0.023) and perceived impact (*p* = 0.036), where residents consistently report scores clustered toward the upper bound of the scale compared to the broader dispersion observed among attendings.

Subgroup comparisons by professional role, years of experience, specialty, and prior app use were exploratory and hypothesis-generating; given the sample size and the number of comparisons performed, they were not powered to confirm differences and should be interpreted as preliminary. Exploratory analysis of SUS and uMARS scores relative to professional characteristics revealed that attendings and residents differed significantly (*p* < 0.05) in median scores for objective and subjective quality domains, as well as the impact in the uMARS (Table 6, Figure 5 and Figure 6). A similar trend appeared when comparing uMARS results by years of experience, contrasting junior (<5 years) vs. senior (≥5 years) clinicians; however, this disparity did not reach statistical significance (Supplementary Material, Tables S1–S3; Figures S2–S10).

**Figure 7:**
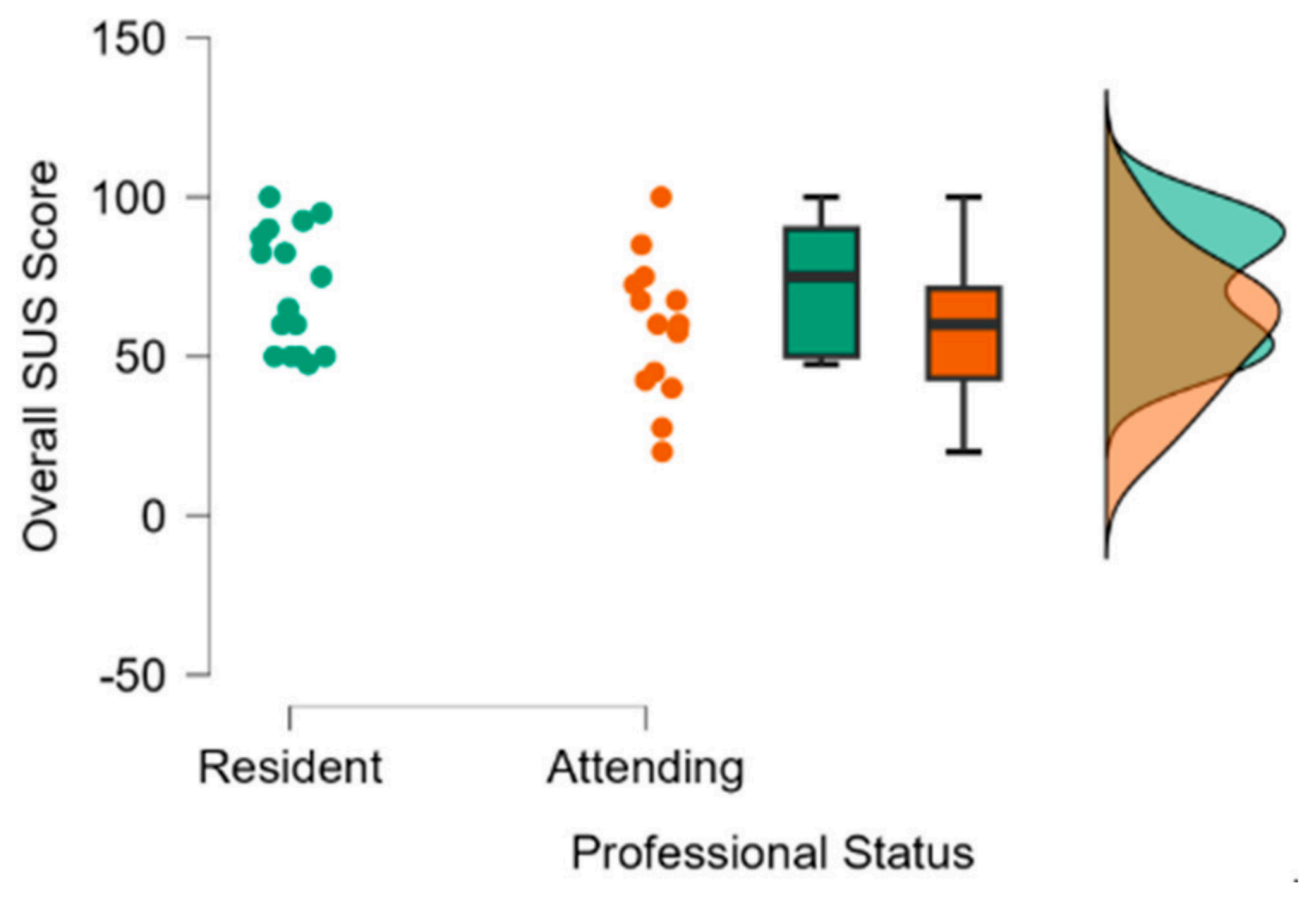
Raincloud plot visualization of the overall System Usability Scale (SUS) scores by professional role. The plot illustrates the distribution and density of usability scores for residents and attendings. Note the overlap between the two groups’ distributions. No statistically significant difference was detected between residents and attendings (*p* = 0.072).

Regarding the SUS, residents reported higher scores than attendings; however, no statistically significant difference was detected (*p* = 0.072) (Figure 7). No statistically significant differences were detected for other variables either, such as prior CDSS use or medical specialty. Given the exploratory nature and limited sample size of this analysis, these results should not be interpreted as evidence that usability perception is equivalent across professional roles, prior CDSS experience, or specialty, but rather that this study was unable to detect a significant difference for these variables.

One possible theoretical explanation for the scoring disparity between residents and attendings is the “expertise reversal effect”, although the present exploratory design does not allow this mechanism to be directly tested or confirmed. This cognitive psychology principle postulates that instructional support (highly effective for novices) loses efficacy or yields negative results in experts. Rooted in cognitive load theory, this suggests that experts possess consolidated mental schemas for near-automatic problem-solving, while novices require external scaffolding to compensate for limited executive function. Providing the same level of assistance to an expert could therefore create a conflict with their prior knowledge base, resulting in extraneous cognitive load due to information redundancy [43]. However, this study did not directly measure cognitive load, mental schema activation, or information redundancy; the applicability of this framework to our findings is therefore inferential and remains to be empirically tested.

This phenomenon is well-documented; Zhang et al. demonstrated that usability fault detection is directly proportional to expertise. While novices identified only 22% of issues, clinical experts detected 41%, and “double experts” (possessing both clinical and systems expertise) reached a 60% detection rate [37]. Furthermore, Newton et al. observed that junior professionals and nursing staff tend to provide more favorable ratings than senior physicians [41].

These findings could suggest that attendings apply a higher evaluative rigor, potentially contrasting UCISAB’s design with consolidated internal mental schemas to identify barriers less apparent to trainees; however, this interpretation remains speculative given the exploratory, cross-sectional design of the study. While this disparity requires further validation through high-powered studies, it identifies a critical research gap. Future studies specifically designed to test the expertise reversal effect in CDSS evaluation—incorporating validated cognitive load measures and larger, stratified samples—are needed to confirm or refute this hypothesis in high-complexity clinical settings. 

## 5. Limitations

While the sample size (*n* = 31) is adequate for frequency description and non-parametric comparative analysis, it possesses limited statistical power for performing robust correlation analyses or multivariate modeling. Therefore, the results of the exploratory analysis regarding the disparity between residents and attendings should be interpreted as hypothesis-generating, requiring validation in larger-scale studies. In addition, several subgroup comparisons were performed across professional role, years of experience, specialty, and prior use of medical applications without correction for multiple testing, which increases the probability of type I error; the differences observed should therefore be regarded as exploratory signals rather than as confirmed effects.

Second, the evaluation was conducted remotely in a controlled, simulated environment, which may limit the ecological validity of the findings. In real-world intensive care unit (ICU) settings, factors such as clinical workload, acute stress, and constant interruptions could yield system interactions different from those reported under simulation. Accordingly, this study evaluated perceived usability and quality only; clinical effectiveness, medication errors, adverse events, and patient outcomes were not assessed, and no patient was exposed to the recommendations generated by the system. The accuracy of the recommendations generated by UCISAB was not objectively validated against a reference standard or an independent expert panel, since the SUS and uMARS instruments capture user-perceived usability and quality rather than algorithmic performance. Likewise, the figure reported for willingness to use reflects a hypothetical decision in a simulated scenario and not actual adoption of the system in clinical practice. Future work should therefore include situated usability testing, carried out in situ at the bedside in the intensive care unit, in order to assess how interruptions, acute stress, and actual clinical workload affect interaction with the system.

Third, participants were free to complete the evaluation using either the mobile or desktop version of UCISAB, and the specific platform used by each participant was not recorded. As interface ergonomics may differ between platforms, this uncontrolled variability could have contributed to the dispersion observed in the usability scores and precludes any analysis of a potential platform effect on usability perception. Future studies should record and, ideally, standardize the platform used.

Fourth, all participants received a synchronous induction session covering the architecture of UCISAB, its supporting clinical evidence, and the completion of the instruments before the evaluation, and all sessions were supervised in real time. Although this procedure standardized exposure across the sample, it may have influenced the usability ratings, since the tool was not assessed under conditions of first, unassisted contact. The reported scores should therefore be interpreted as usability after structured training rather than as spontaneous learnability.

Fifth, the qualitative thematic analysis was performed by a single coder, without independent double-coding. Although this is consistent with the reflexive thematic analysis framework adopted, in which analytic subjectivity is considered a resource rather than a limitation, the absence of a second coder may still have introduced interpretative bias; future studies incorporating multiple coders could further strengthen the robustness of the thematic findings.

Sixth, the generalizability of these findings is limited. The evaluation was carried out in two centers in a single country, using a non-probabilistic convenience sample recruited in part through peer referral, and was restricted to physicians. Other professionals directly involved in the management of sedation and analgesia in the intensive care unit, particularly nurses, respiratory therapists, and clinical pharmacists, were not included, so their usability requirements remain unexplored. The results may therefore not be transferable to other settings, health systems, or professional groups.

Finally, the voluntary nature of participation may have introduced selection bias, potentially recruiting professionals with an inherent positive predisposition toward digital health technologies.

## 6. Conclusions

In this evaluation, conducted in a controlled and simulated environment, UCISAB was perceived by the participating physicians as a clinical decision support system (CDSS) of high technical and informational quality, while its usability score remained slightly below the accepted threshold. This pattern suggests that the perceived value of a CDSS in high-complexity environments may depend not only on the content of its recommendations but also on an interface architecture that minimizes cognitive load and synchronizes with the sequential workflow of critical care. The accuracy of the recommendations, the clinical effectiveness of the system, and its safety were not evaluated in this study.

Furthermore, this research points to the “expertise reversal effect” as a hypothesis worth examining in health technology assessment. The perceptual divergence observed between residents and specialists, which requires confirmation in adequately powered studies, suggests that the design of future tools should be adaptive, offering differentiated guidance levels based on the end-user’s mental schema.

Finally, the high stated willingness to use suggests that tools of this type respond to a need perceived by the clinicians who evaluated the system. Whether UCISAB is actually adopted, and whether it improves clinical processes or patient outcomes, remains to be determined in studies conducted with real patients, following refinement focused on informational transparency and visual ergonomics. Overall, UCISAB showed favorable perceived quality and high stated willingness to use, but usability remained marginal; further interface and pharmacological refinements and prospective clinical validation in real-world settings are required before routine implementation.

## Figures and Tables

**Table 1:** Sociodemographic and professional characteristics of the participating physicians (*n* = 31).

Variable	Frequency (n)	Percentage (%)
Gender		
Female	16	51.6
Male	15	48.4
Professional Role		
Resident	17	54.8
Attending	14	45.2
Specialty		
Critical Care	22	71.0
Internal Medicine	5	16.1
Anesthesiology	2	6.5
General Surgery	1	3.2
Clinical Pharmacology	1	3.2
Professional Experience		
1 to 2 years	7	22.6
3 to 5 years	11	35.5
6 to 10 years	7	22.6
More than 10 years	6	19.4
Prior Use of Medical Applications		
No	20	64.5%
Yes	11	35.5%
Age (years)	Mean 34.7	SD: ± 7.3

**Table 2:** Internal consistency analysis of the scales used (*n* = 31).

Instrument/Dimension	N° of items	Cronbach’s Alpha (α)	95% CI [LB–UB]
SUS (Global)	10	0.883	0.832–0.934
uMARS (Global Objective Quality)	16	0.943	0.919–0.967
Section A: Engagement	5	0.865	0.785–0.944
Section B: Functionality	4	0.854	0.772–0.937
Section C: Aesthetics	3	0.889	0.822–0.955
Section D: Information	4	0.840	0.708–0.972

**Table 3:** Evaluation of the UCISAB application quality using the uMARS (*n* = 31).

uMARS Dimension	Median (1–5)	IQR (P25–P75)
Objective Quality		
Section A: Engagement	4.20	3.30–4.60
Section B: Functionality	4.25	3.75–4.75
Section C: Aesthetics	4.33	3.33–4.67
Section D: Information	4.50	4.00–5.00
Total Objective Quality	4.28	3.57–4.63
Subjective Quality		
Section E: Subjective Quality	3.75	3.12–4.12
Acceptability: Adoption **	4.00	3.50–5.00
Acceptability: Recommendation **	4.00	4.00–5.00
User-Perceived Impact		
Section F: Perceived Impact	4.33	4.00–5.00

**Notes:** Values are represented as median and IQR (interquartile range). The maximum possible score for each dimension is 5.0. ** The specific subjective quality items described are part of the acceptability evaluation according to the methodology described by ref. [32].

**Table 4:** Evaluation of the UCISAB application usability using the SUS (*n* = 31).

System Usability Scale (SUS) Results	Mean (0–100)	SD (±)
Global Score	66.13	21.26

**Table 5:** Thematic analysis of feedback from UCISAB CDSS evaluators.

Theme	Codes	Representative Quotes
**Clinical Utility**	Practice standardization	“Important specific recommendations for sedoanalgesia weaning” (Att. P13) “Facilitates the medical order” (Res. P1) “I find it very useful for identifying withdrawal risk and its management” (Res. P33) “Sedoanalgesia calculation is achieved quickly and practically” (Res. P2)
	Scientific evidence base	“It is useful, evidence-based” (Att. P5) “Referenced information” (Att. P17) “I liked that it offers concrete information” (Res. P3)
**UI-UX Challenges**	Visual design and semantics	“Visually unattractive” (Att. P4) “It seems visually boring to me” (Att. P18) “Could have a slightly more visually friendly interface” (Res. P29) “Opportunities: more didactic icons” (Res. P34) “It would be appropriate to include icons, shapes, and symbols to differentiate the options” (Res. P3)
	Navigation flow	“If the interface were one element after another, instead of just tabs, it would be more intuitive and simpler” (Att. P5) “Data entry should be better signaled” (Att. P17) “There is no clarity in the arrows at the top to enter all data” (Att. P17) “The interface for selecting the patient can be tedious” (Att. P37) “In the sedation calculation, it doesn’t allow for easy dose modification” (Att. P37)
	Perceived complexity	“Very complex and not very intuitive to use” (Att. P18) “The box requesting sedation targets is not very clear” (Att. P19) “Requires an explanation to be used” (Att. P4) “It’s not that intuitive, and the initial menu is presented in a flat way” (Res. P3)
	Workflow disconnection	“No continuity is evident in the data entered across different tabs, making the initial doses feel unnecessary” (Att. P7) “They give standard doses at the start and then standard weaning based only on risk, making data and steps feel disconnected and unnecessary” (Att. P7) “Infusion cc/hour is not useful” (Att. P6)
**Algorithmic Refinement and Pharmacological Depth**	Score interpretation	“Does not adequately explain withdrawal risks” (Att. P6) “It should explain why it recommends that specific methadone dose and the conversion.” (Res. P33) “The CPOT scale only shows the score […] I consider score interpretation necessary.” (Res. P2)
	Clinical formulary integrity and updates	“Include dexmedetomidine” (Att. P32) “Most frequently used Propofol presentations: 20 mL (200 mg), 50 mL (500 mg)” (Att. P32) “Does not mention cumulative opioid doses; does not differentiate withdrawal between opioids and benzodiazepines” (Att. P6) “Consider if there is more than one sedation agent, e.g., midazolam and propofol, as that would affect the doses” (Att. P32) “Recommended doses differ from real scenarios and current literature” (Att. P6)

**Note:** Att = attending; Res = resident; P = participant. **Question:** *“If the simulated patients were real, would you apply the results obtained in clinical practice?”*

**Table 6:** Comparison of usability perception (SUS) and quality dimensions (uMARS) of UCISAB by the evaluators’ professional role (*n* = 31).

Outcome Variable	Residents (*n* = 17)	Attendings (*n* = 14)	*p*-Value	Effect Size (95% CI)
SUS score (SD)	72.35 (±19.07)	58.57 (± 21.99)	0.072	d = 0.67 (−0.06 to 1.48)
uMARS Objective Quality (Median [IQR])	4.59 (4.41–4.84)	3.43 (3.18–4.27)	<0.001	r = 0.75 (0.50–0.88)
Section A: Engagement	4.60 (4.20–4.80)	3.30 (2.85–3.95)	<0.001	r = 0.70 (0.43–0.86)
Section B: Functionality	4.50 (4.25–4.75)	3.75 (2.87–4.25)	0.003	r = 0.63 (0.32–0.82)
Section C: Aesthetics	4.67 (4.33–5.00)	3.50 (3.00–4.33)	0.004	r = 0.61 (0.28–0.81)
Section D: Information	4.75 (4.50–5.00)	4.12 (3.75–4.44)	0.002	r = 0.65 (0.35–0.83)
uMARS Subjective Quality (Median [IQR])	4.00 (3.50–4.25)	3.12 (2.56–4.00)	0.023	r = 0.48 (0.11–0.74)
uMARS Impact (Median [IQR])	5.00 (4.17–5.00)	4.17 (3.87–4.59)	0.036	r = 0.44 (0.05–0.71)

**Notes:** *p*-values were calculated using Student’s *t*-test for the SUS and the Mann–Whitney U test for the uMARS. Effect size is expressed as Cohen’s d for the SUS and as rank-biserial correlation (r) for the uMARS dimensions, with 95% confidence intervals; all effect sizes are reported as absolute values, with higher scores favoring residents in all comparisons. SD: standard deviation; IQR: interquartile range.

## Data Availability

The anonymized datasets generated and analyzed during this study, comprising the System Usability Scale and user version of the Mobile Application Rating Scale responses, are available from the corresponding author upon reasonable request.

## Supplementary Materials

The following supporting information can be downloaded at https://www.mdpi.com/article/10.3390/jcm15187191/s1, Table S1: Comparison of usability perception (SUS) and quality dimensions (uMARS) of UCISAB by years of professional experience; Table S2: Comparison of usability perception (SUS) and quality dimensions (uMARS) of UCISAB by evaluator specialty; Table S3: Comparison of usability perception (SUS) and quality dimensions (uMARS) of UCISAB by the evaluators’ prior clinical app usage; Figure S1: Kernel density analysis for the global usability (SUS) and objective technical quality (uMARS) scores; Figure S2: Raincloud plot visualization of overall System Usability Scale (SUS) scores by years of clinical experience; Figure S3: Raincloud plot visualization of the overall objective quality (uMARS) by years of clinical experience; Figure S4: Raincloud plot visualization of (A) subjective quality and (B) perceived impact scores (uMARS) by years of clinical experience; Figure S5: Raincloud plot visualization of the overall usability (SUS) scores by evaluator specialty; Figure S6: Raincloud plot visualization of the overall objective quality (uMARS) by evaluator specialty; Figure S7: Raincloud plot visualization of (A) subjective quality and (B) perceived impact scores (uMARS) by evaluator specialty; Figure S8: Raincloud plot visualization of the overall usability (SUS) scores by prior clinical app usage; Figure S9: Raincloud plot visualization of the overall objective quality (uMARS) by prior clinical app usage; Figure S10: Raincloud plot visualization of (A) subjective quality and (B) perceived impact scores (uMARS) by prior clinical app usage. Supplementary Material Section S1: Clinical Algorithms of UCISAB (Table S4: Sedative infusion rates by target RASS level; Table S5: IOWS risk classification matrix and corresponding system action); Supplementary Material Section S2: Simulated Clinical Cases Used for the UCISAB Evaluation.
