# Supplementary material for: Usability and Quality of UCISAB, a Clinical Decision Support System for Optimization of Analgesia and Sedation Infusions in the ICU: A Multicenter, Cross-Sectional, Mixed-Methods Usability Evaluation in Colombia
Source: J Clin Med. 2026 Sep 16;15(18):7191. doi: 10.3390/jcm15187191 (PMC13607049; doi:10.3390/jcm15187191)
Supplement: Supplementary file 1 [file jcm-15-07191-s001.zip › jcm-4523342-supplementary.pdf]

Usability and Quality of UCISAB, a Clinical Decision Support System for Analgesia and Sedation Infusion Optimization in the ICU: A Multicentric Evaluation in Colombia.

Supplementary Material

Table S1. Comparison of usability perception (SUS) and quality dimensions (uMARS) of UCISAB by years of professional experience *(n=31)*.

Outcome Variable	Junior (n=18)	Senior (n=13)	P – value	Effect size (95% CI)
**SUS score (SD)**	68.0 (±17.85)	63.5 (± 25.79)	0.562	d = 0.21 (-0.50 to 0.93)
**uMARS Objective Quality (Median [IQR])**	4.57 (3.09 – 4.66)	3.79 (3.27 – 4.37)	0.057	r = 0.41 (-0.02 to 0.69)
**uMARS Subjective Quality (Median [IQR])**	4.00 (3.31 – 4.18)	3.50 (2.75 – 4.00)	0.323	r = 0.21 (-0.20 to 0.56)
**uMARS Impact (Median [IQR])**	4.58 (4.17 – 5.00)	4.17 (3.83 – 5.00)	0.156	r = 0.30 (-0.11 to 0.62)

Notes: *Junior is defined as < 5 years of experience and Senior as ≥ 5 years. P-values were calculated using Student's t-test for the SUS scale and the Mann-Whitney U test for the uMARS scale. Effect size is expressed as Cohen's d for the SUS and rank-biserial correlation (r) for uMARS dimensions, with signs reflecting the group coding. As none of these comparisons reached statistical significance, directionality is not interpreted. SD: Standard Deviation; IQR: Interquartile Range; CI: Confidence Interval.*

Table S2. Comparison of usability perception (SUS) and quality dimensions (uMARS) of UCISAB by evaluator specialty *(n=31)*.

Outcome Variable	Critical Care Specialty (n=22)	Other Specialties (n=9)	P – value	Effect size (95% CI)
**SUS score (SD)**	67.16 (±22.31)	63.61 (± 19.45)	0.681	d = 0.16 (-0.61 to 0.94)
**uMARS Objective Quality (Median [IQR])**	4.28 (3.41 – 4.60)	4.58 (3.79 – 4.84)	0.296	r = 0.25 (-0.20 to 0.61)
**uMARS Subjective Quality (Median [IQR])**	3.62 (3.06 – 4.00)	4.00 (3.50 – 4.25)	0.430	r = 0.19 (-0.26 to 0.57)
**uMARS Impact (Median [IQR])**	4.33 (4.00 – 5.00)	4.33 (4.17 – 5.00)	0.806	r = 0.06 (-0.38 to 0.47)

Notes: *P-values were calculated using Student's t-test for the SUS scale and the Mann-Whitney U test for the uMARS scale. The specialty variable was dichotomized: "Other Specialties" includes internal medicine, general surgery, anesthesiology, and clinical pharmacology. Effect size is expressed as Cohen's d for the SUS and rank-biserial correlation (r) for uMARS dimensions, with signs reflecting the group coding. As none of these comparisons reached statistical significance, directionality is not interpreted. SD: Standard Deviation; IQR: Interquartile Range; CI: Confidence Interval.*

Table S3. Comparison of usability perception (SUS) and quality dimensions (uMARS) of UCISAB by the evaluators' prior clinical app usage *(n=31)*.

Outcome Variable	No Prior App Usage (n=20)	Prior App Usage (n=11)	P – value	Effect size (95% CI)
**SUS score (SD)**	65.50 (±23.71)	67.27 (± 16.90)	0.829	d = -0.08 (-0.82 to 0.65)
**uMARS Objective Quality (Median [IQR])**	4.27 (3.36 – 4.59)	4.57 (3.88 – 4.73)	0.311	r = 0.23 (-0.20 to 0.58)
**uMARS Subjective Quality (Median [IQR])**	4.00 (2.75 – 4.06)	3.50 (3.25 – 4.12)	0.884	r = 0.04 (-0.38 to 0.44)
**uMARS Impact (Median [IQR])**	4.33 (4.00 – 5.00)	4.33 (4.08 – 4.75)	0.718	r = - 0.08 (-0.47 to 0.34)

Notes: *P-values were calculated using Student's t-test for the SUS scale and the Mann-Whitney U test for the uMARS scale. Effect size is expressed as Cohen's d for the SUS and rank-biserial correlation (r) for uMARS dimensions, with signs reflecting the group coding (No vs. Prior app usage). As none of these comparisons reached statistical significance, directionality is not interpreted. SD: Standard Deviation; IQR: Interquartile Range; CI: Confidence Interval.*

Images:

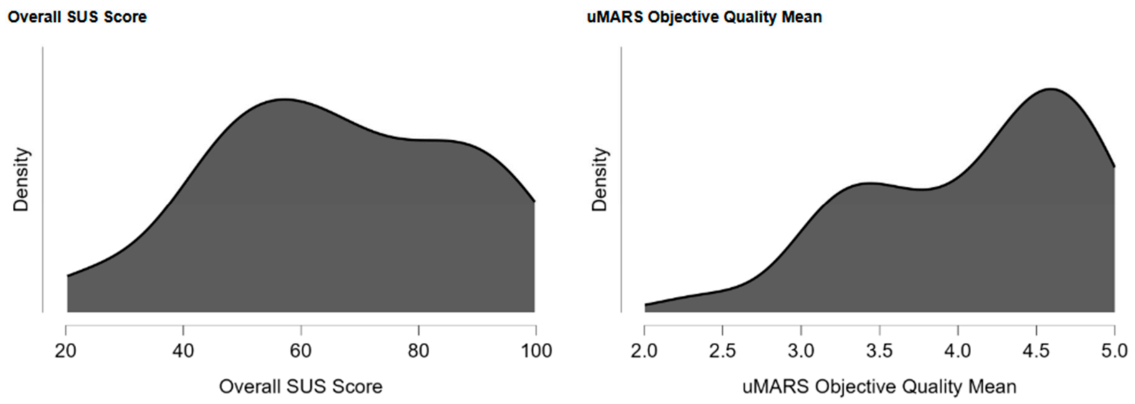

Figure S1. Kernel density analysis for the global usability (SUS) and objective technical quality (uMARS) scores. The distribution morphology is presented for the entire evaluator sample *(n = 31)*. For the SUS scale, the curve displays an approximately symmetric distribution, which visually supports the application of parametric tests. Conversely, the uMARS scale exhibits a negatively skewed distribution, confirming the non-parametric nature of this variable.

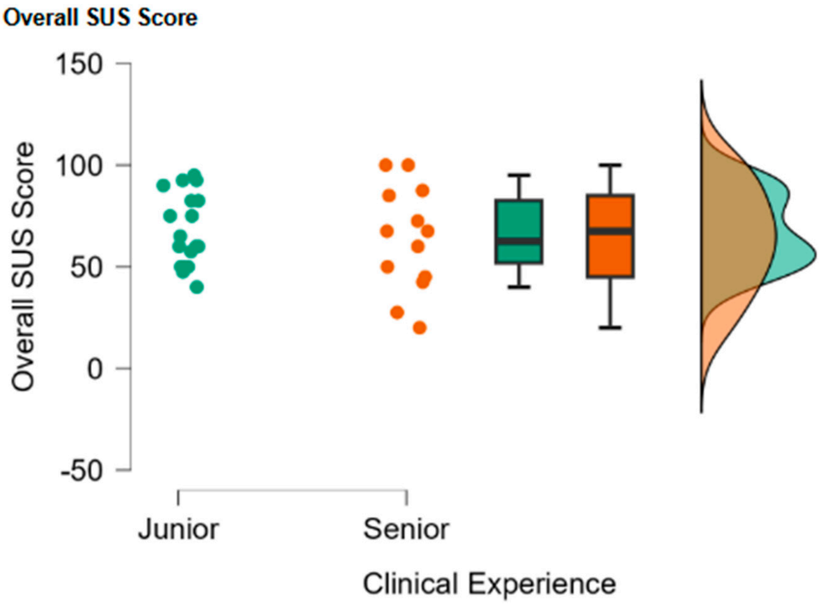

Figure S2. Raincloud plot visualization of overall System Usability Scale (SUS) scores by years of clinical experience. The plot displays the score distribution for Junior (< 5 years) and Senior (≥ 5 years) evaluators. No statistically significant difference was detected ($p = 0.562$).

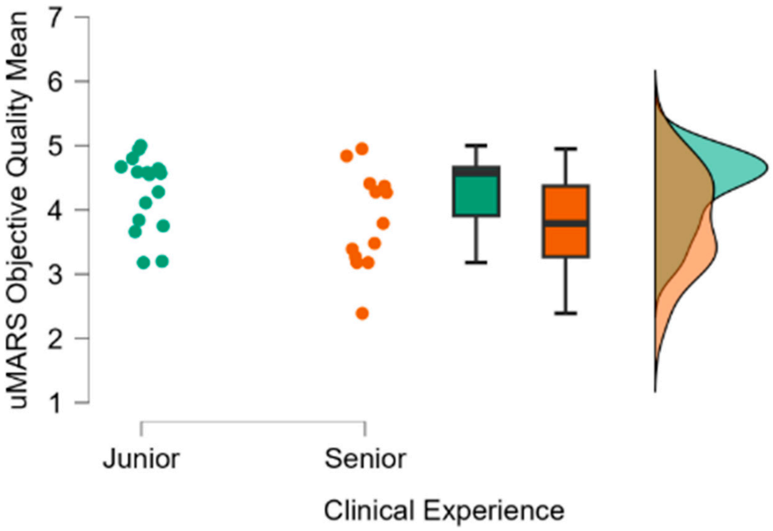

Figure S3. Raincloud plot visualization of the overall objective quality (uMARS) by years of clinical experience. Note the slight trend toward higher scores in the Junior group (p = 0.057), though it does not reach statistical significance.

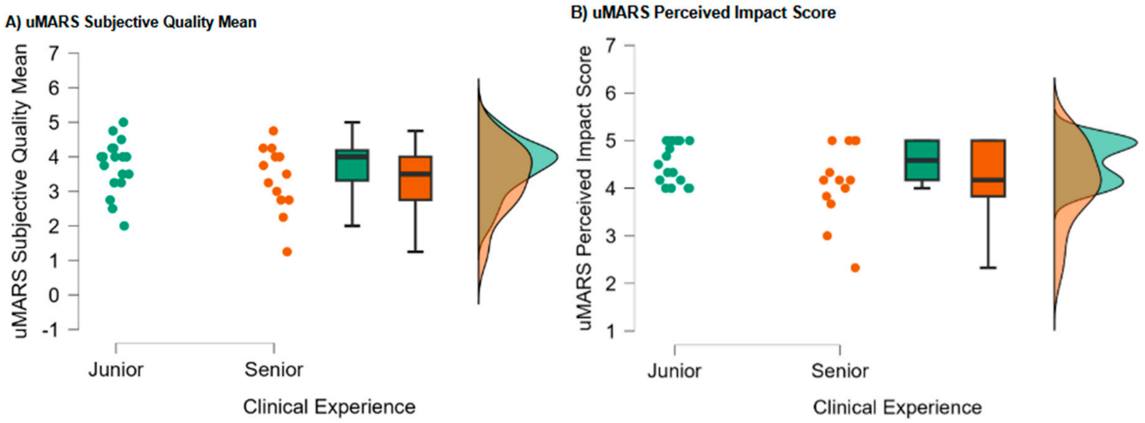

Figure S4. Raincloud plot visualization of (A) subjective quality and (B) perceived impact scores (uMARS) by years of clinical experience.

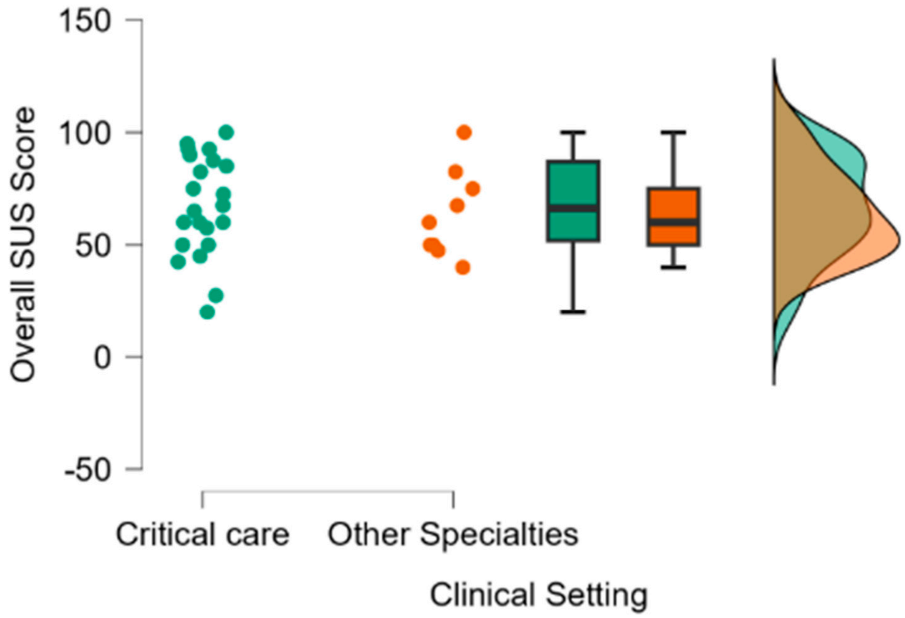

Figure S5. Raincloud plot visualization of the overall usability (SUS) scores by evaluator specialty.

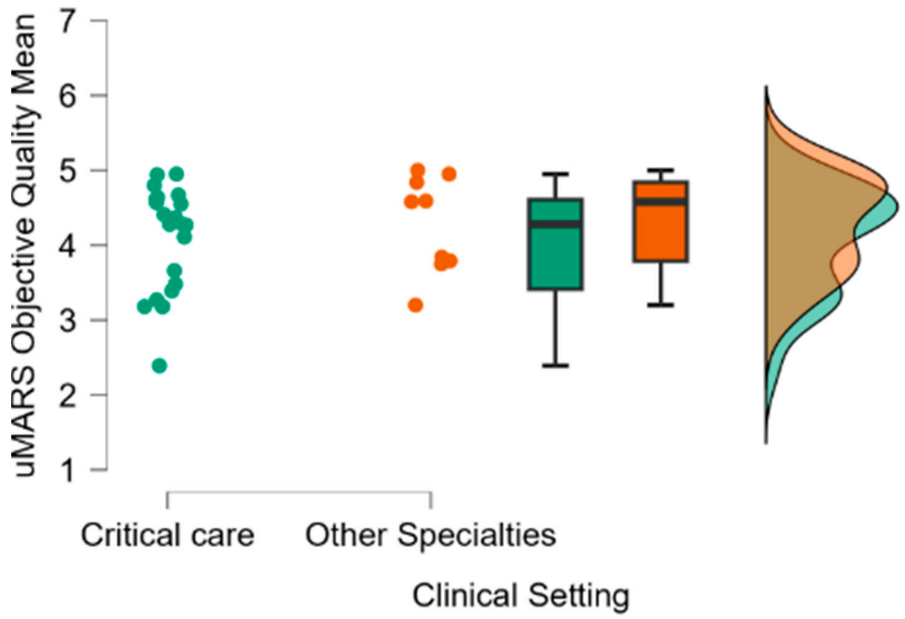

Figure S6. Raincloud plot visualization of the overall objective quality (uMARS) by evaluator specialty.

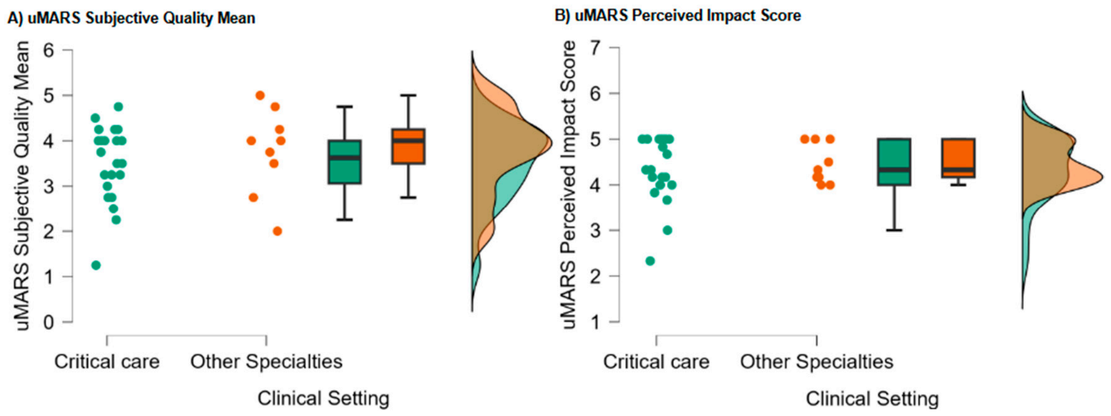

Figure S7. Raincloud plot visualization of (A) subjective quality and (B) perceived impact scores (uMARS) by evaluator specialty.

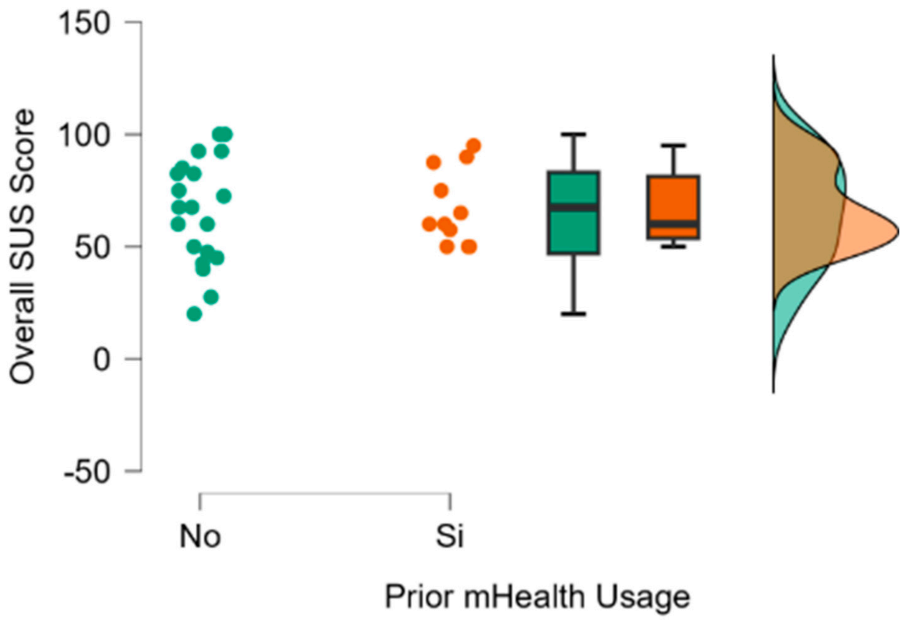

Figure S8. Raincloud plot visualization of the overall usability (SUS) scores by prior clinical app usage.

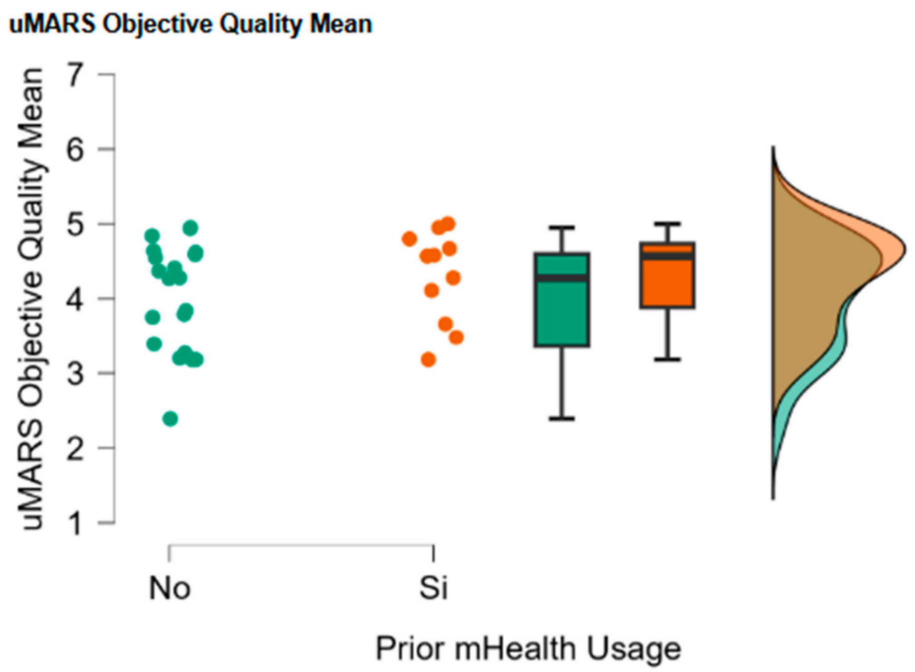

Figure S9. Raincloud plot visualization of the overall objective quality (uMARS) by prior clinical app usage.

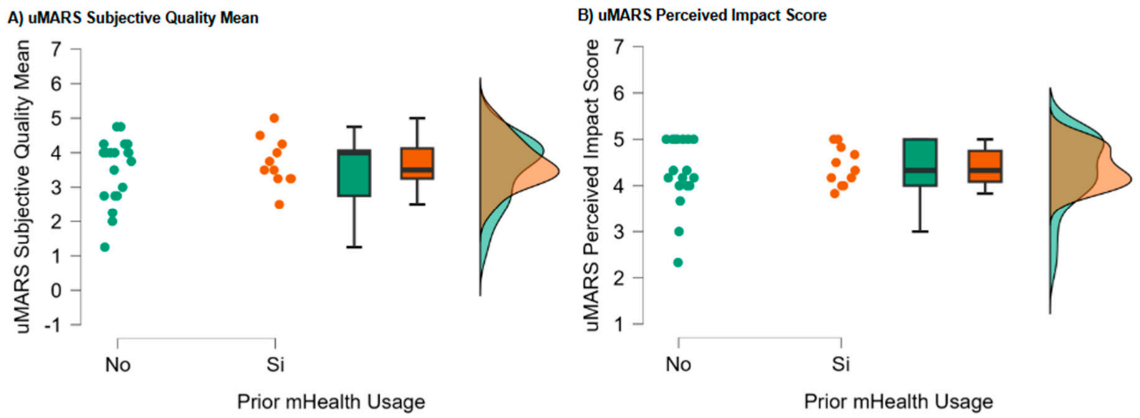

Figure S10. Raincloud plot visualization of (A) subjective quality and (B) perceived impact scores (uMARS) by prior clinical app usage.

Suppl. Material S1: Clinical Algorithms of UCISAB

The version of UCISAB evaluated in this study corresponds to a minimum viable product. Algorithms were implemented for the three sedatives most frequently used in the participating intensive care units (midazolam, propofol, and ketamine) and for fentanyl, the opioid most frequently used in this setting. Dose ranges were taken from Devlin et al.; within those ranges, the specific value assigned to each RASS and CPOT level was defined pragmatically by author consensus for this prototype. The module operates as a calculator intended to provide a supporting margin, and the final dose remains a decision of the treating physician.

Table S4. Sedative infusion rates by target RASS level

RASS level	Midazolam (mg/kg/h)	Propofol (mg/kg/h)	Ketamine (mg/kg/h)
Alert and calm (0)	0	0	0
Drowsy (−1)	0.04	0.3	1
Light sedation (−2)	0.06	0.8	1.1
Moderate sedation (−3)	0.08	1.3	1.2
Deep sedation (−4)	0.1	2	1.5
Unarousable (−5)	0.12	4	2

Analgesia is titrated to the Critical-Care Pain Observation Tool (CPOT) score, using fentanyl at 1 µg/kg/h for mild pain (CPOT below 2), 3 µg/kg/h for moderate pain (CPOT 2 to 4), and 5 µg/kg/h for severe pain (CPOT above 4).

The rules of the iatrogenic opioid withdrawal syndrome (IOWS) module were adapted from evidence syntheses and published protocols. Five clinical variables are used to classify the patient into one of four risk levels: duration of opioid infusion (less than 7 days or 7 days or more), presence of risk factors for iatrogenic withdrawal, presence of two or more withdrawal signs, a RASS score of 2 or higher, and a CPOT score of 2 or higher. Withdrawal signs were assessed using the DSM-5 criteria adapted to the local context.

Table S5. IOWS risk classification matrix and corresponding system action

Risk level	Days on opioids	Clinical conditions ("yes" answers)	System action
No risk	Less than 7	None (all other variables "no")	General recommendations only
Low risk	Less than 7	One variable altered: IOWS risk factors, or RASS, or CPOT	Low-risk alert; discharge planning allowed
Low risk	7 or more	None (all other variables "no")	Low-risk alert; discharge planning allowed

High risk	Less than 7 or 7 or more	Any other combination not described in this table (e.g. IOWS signs plus RASS at less than 7 days, or risk factors plus CPOT at 7 days or more)	High-risk alert
Very high risk	Less than 7	All four clinical variables altered (risk factors, signs, RASS and CPOT)	Very-high-risk alert; discharge blocked or conditioned
Very high risk	7 or more	Withdrawal signs combined with RASS, CPOT or risk factors	Very-high-risk alert

Tapering recommendations by risk level

- No risk: the infusion may be discontinued directly.
- Low risk: reduce the dose by 50% every 6 hours; fentanyl rescue doses of 1–2 µg/kg may be used.
- High risk: reduce the dose by 30% every 6 hours, with fentanyl rescues and the option of methadone (10 mg every 6 hours) or clonidine, or resumption at 80% of the previous rate if symptoms reappear at reassessment.
- Very high risk: reduce the dose by 30% every 12 hours, with methadone (10 mg every 6 hours), clonidine, or buprenorphine at an initial dose of 0.6 mg/day; a 20% reduction every 24 hours or a return to 80% of the previous dose may also be considered if IOWS is confirmed.

These recommendations were defined by author consensus on the basis of the sources cited in the manuscript. No formal external validation of the algorithm against a reference standard was carried out prior to the usability evaluation.

Suppl. material S2: Simulated Clinical Cases Used for the UCISAB Evaluation

The following two standardized simulated clinical cases were presented, in fixed order, to all participants. Both cases were identical across the sample. Sessions were supervised in real time (in person or remotely) by a member of the research team, who clarified questions regarding the operation of UCISAB and the completion of the SUS and uMARS scales. The complete case session lasted approximately 30 minutes on average. Fictitious patient identifiers were used for record creation within the application.

Case # 1

Module: Sedoanalgesia Calculator

A 44-year-old male patient, weighing 88 kg and 1.68 m tall (BMI: 31.2 kg/m^2), was admitted to the ICU 24 hours ago with a diagnosis of Acute Respiratory Distress Syndrome (ARDS) secondary to influenza pneumonia. His only relevant medical history is hypercholesterolemia managed with atorvastatin 20 mg every 24 hours; he denies prior opioid use. During his ICU stay he develops respiratory deterioration and requires invasive mechanical ventilation. Following intubation, the Critical-Care Pain Observation Tool (CPOT) yields a score of 1 (controlled pain). Once the airway is secured, the physician must indicate the sedoanalgesia infusion to be used.

- Given the indication for mechanical ventilation, deep sedation is sought (a Richmond Agitation Sedation Scale [RASS] score of -4 to -5); select the medications to be used in this scenario according to your clinical judgment.
- Using the UCISAB app, enter the data and review the dose recommendations generated by the application.
- You may select the ampoule presentation of the medications, the number of ampoules to use, and the solution volume, according to the practice at your institution. Finally, review the conversion the application performs from mcg/h to cc/h (note that the application will base the calculation on the customized dilution).
- Note any opportunities for improvement or positive aspects you identify on a sheet, to later describe them briefly in the survey response.

Case # 1 (continued) — Module: Sedoanalgesia Tapering

Eight days after intubation, the patient meets ventilatory and blood-gas criteria for extubation. On review, the patient has a fentanyl infusion at 3 mcg/kg/h (264 mcg/h), started more than 7 days ago. The clinical record notes that over the past 48 hours the patient develops dysautonomia whenever the infusion is decreased, presenting tachycardia, diaphoresis, piloerection, and diarrhea (note that the patient has risk factors for iatrogenic opioid withdrawal syndrome due to the cumulative dose received and obesity). Facial tension, slow limb movements, and muscle rigidity are observed, but with adequate ventilator synchrony, a CPOT score of 3, and a RASS of +2 was calculated.

- Please enter the data into the UCISAB application and review the recommendations it generates. Based on your judgment, select which you would adopt in a real scenario for a patient with these characteristics.

- Note any opportunities for improvement or positive aspects you identify on a sheet, to later describe them briefly in the survey response.

Case # 2

Module: Sedoanalgesia Tapering

A 65-year-old female patient, weighing 80 kg and 1.58 m tall (BMI: 32 kg/m²), with a history of herniated disc and chronic oxycodone use, is admitted to the hospital for abdominal sepsis secondary to cholangitis. The patient develops clinical deterioration and has required mechanical ventilation for 4 days; however, with antibiotic and endoscopic management she improves, meeting ventilatory and blood-gas criteria for extubation. She currently has a fentanyl infusion at 2 mcg/kg/h (160 mcg/h), with a RASS of −2. On review of the clinical record and physical examination, no signs of Iatrogenic Opioid Withdrawal Syndrome are found, but risk factors for developing it are present due to elevated BMI and chronic opioid use; finally, there are no signs of pain, with a CPOT of 0.

- Please enter the data into the UCISAB application and review the recommendations it generates. Based on your judgment, select which you would adopt in a real scenario for a patient with these characteristics.
- Note any opportunities for improvement or positive aspects you identify on a sheet, to later describe them briefly in the survey response.
